# Lasers in gynaecology – Are they still obsolete? Review of past, present and future applications *

**Published:** 2020-05-07

**Authors:** ESGE Special Interest Group ‘Innovations’ Working Group, U Catena, A Rosati, MM Ianieri, I Romito, N Vlahos, A Daniilidis, G Scambia, S Becker

**Affiliations:** Catholic University of the Sacred Heart, Rome, Italy;; Fondazione Policlinico Universitario A. Gemelli IRCCS, Roma, Italy;; Second Department of Obstetrics and Gynaecology, Aretaieion University Hospital, University of Athens School of Medicine, Athens, Greece;; Second Department of Obstetrics and Gynaecology, Hippokratio General Hospital Aristotle University, Thessaloniki, Greece;; Department of Gynaecology and Obstetrics, University Hospital Frankfurt, Frankfurt, Germany.

**Keywords:** Laser, laparoscopy, hysteroscopy, endometriosis

## Abstract

After the advent and the subsequent gradual disuse of laser technology in gynaecological surgery, in recent years, thanks to technical improvements, this technology is progressively reaffirming itself in various areas of minimally invasive gynaecological surgery ranging from laparoscopy to robotic surgery and hysteroscopy.

This paper, through a SWOT (strengths, weaknesses, opportunities and threats) analysis, shows positives and negatives of this technology with particular attention to present and future applications.

## Background

Laser (Light Amplification by Stimulated Emission of Radiation) is a useful energy source available for gynaecological endoscopy. Laser technology is based on the amplification of a specific light wavelength that generates the emission of a beam of photons with a high degree of spatial and temporal coherence. The contact of a laser beam with organic tissue generates molecular vibration, inducing the disruption of chemical bonds and the production of heat. Tissue effect is the result of laser beam absorption, refraction and reflection and can be modulated by adapting exposure time and power density (Law et al., 2014).

The selective tissue absorption characteristics of different kind of lasers can be exploited in surgery. The CO_2_ laser is excellent for tissue vaporisation because it is absorbed by water but is not effective in tissue desiccation or coagulation; while the Nd:YAG laser could be selectively absorbed by pathologically coloured tissues containing haemoglobin such as endometriotic tissue (Lomano, 1987).

Initially, the use of the laser was promoted as a response to some of electrosurgery complications. Specifically, the laser offered the possibility of more selective tissue effect through minimal lateral thermal damage when compared to classical electrosurgery.

**Figure 1:**
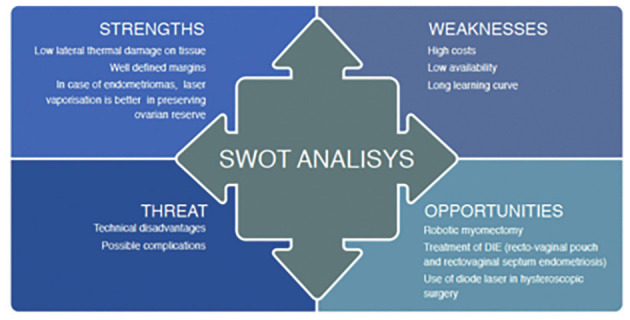
— SWOT Analysis on lasers in gynaecology. DIE: Deeply infiltaring endometriosis.

In this regard, the following SWOT analyses will focus on positives and negatives of this technology with particular attention to present and future applications (Figure 1).

## Strengths

Several studies have compared different types of energy employed in gynaecological surgery and their collateral tissue effects. Laser energy inflicts the least amount of damage to the surrounding tissues both in human and animal models and it shows greater surgical precision than monopolar electrosurgery, both in terms of cut and coagulation modes.

Bailey et al. (2014) have shown, in animal models, how monopolar energy causes a greater collateral thermal damage on the myometrium than the flexible CO_2_ laser fibre. This lateral thermal damage increases in a linear way with monopolar power settings. Conversely increasing the power setting of the laser (in the range from 5 to 15 W) affects only the depth of incision and dissection capacity without increasing collateral thermal damage.

Also in a microscopic comparison of the shape of the incision, the Nd:YAG laser produced the smoothest lesions with well-defined margins compared to the monopolar energy that was more often associated with irregular and fissured margins (Schurr et al., 1994).

Concerning endometriotic cyst management, laparoscopic endometrioma excision was considered the gold standard of care being more effective than drainage or vaporisation in terms of pelvic pain control (Saito et al., 2014), recurrence and pregnancy rate. The pregnancy rate increases after laparoscopic cystectomy, varying from 30% to 67%, with an average of approximately 50% (Vercellini et al., 2009).

In a randomised trial of 90 women comparing ovarian cystectomy and CO_2_ laser vaporisation of the internal wall of the endometrioma, Carmona et al. (2011) found that the laser group was affected by an earlier time of recurrence (7.5 vs 18.1 months, P < 0.003) and a significantly higher rate of recurrence at 12 months’ follow-up (11% vs 31%, P = 0.04).

Conversely, the absence of a clear cleavage plan during stripping determines an involuntary removal of healthy ovarian cortex that is proportional to the size of the cyst itself (Saito et al., 2018).

Several studies show how adverse changes in ovarian vascularisation after stripping (Li et al., 2009; Suksompong et al., 2012) inducing ischemic vascular damage to the residual ovary, can be a consequence of the attempt to reach an accurate haemostasis via bipolar coagulation or through the application of haemostatic stitches on the ovary (La Torre et al., 1998). It has been widely accepted that only the internal lining of the cyst wall should be destroyed with a depth of ablation not exceeding 1.0–1.5 mm, due to endometriotic cells being localised only on the surface of the cyst capsule (Saridogan et al., 2017).

Taking this perspective makes the use of laser ablation of endometriotic cysts interesting: CO_2_ laser ablation was demonstrated to be effective in the treatment of endometriomas in three different settings: the “three stages technique”, the “stripping and ablation combined technique” and the “pure ablative technique”. Tsolakidis et al. (2010) and Pados et al. (2010) performed a randomised controlled trial comparing the stripping technique with the so-called “three-stage technique”. This last procedure consists of: 1) Laparoscopic fenestration and drainage of the endometriotic cyst; 2) Gonadotrophin-releasing hormone agonist (GnRHa) therapy for 3 months 3) second laparoscopy in which CO_2_ laser ablation of the cyst wall is performed. They concluded that ovarian vascularisation and volumes were comparable between the two laparoscopic techniques, but the follicular reserve, determined by AFC (antral follicle count) at six months, was significantly higher in patients subjected to the “three-step procedure” compared to the other group. Furthermore, although the ovarian reserve did not improve in either group, the AMH level is less diminished after the three-step procedure compared with cystectomy.

Donnez et al. (2010) and Nappi et al. (2016) proposed a combined technique, stripping most of the cyst wall and vaporising the remaining 10–20% of endometrioma wall close to the hilum, using the CO_2_ laser or DWLS (new diode laser). Donnez et al. (2010) found a pregnancy rate of 41% at 8.3 months and a recurrence rate of only 2%.

Recently, Munrós et al. (2019) confirmed how ablative techniques, such as CO_2_ laser vaporisation, are better in preserving ovarian reserve compared to laparoscopic stripping by reducing the accidental removal of healthy tissue with a subsequent lower post-surgical inflammatory and procoagulant state whose marker is the curve of microparticles. The increase in the microparticles level is observed only after stripping but not after laser vaporisation, highlighting how this procedure determines a minimal inflammatory response that doesn’t negatively affect the ovarian reserve in terms of AMH and AFC at six months after surgery.

Finally, laser vaporisation shows similar pregnancy rates to cystectomy at long- term follow-up (1 and 5 years): this data has been confirmed by a recent meta-analysis (Dan et al., 2013).

## Weaknesses

Lasers were introduced into gynaecological surgery almost forty years ago; the CO_2_ laser was the first one developed by Patel and his colleagues in 1964 (Bell Laboratories in California) (Sutton et al., 2013).

Laser utilisation experienced the acme in the 1980’s and its use was clinically validated in 1994 with a randomised, double-blind controlled trial for the treatment of pelvic pain associated with endometriosis (Sutton et al., 1994). This period was followed by a progressive decline due to the advent of laparoscopy. In the laparoscopic setting, lasers become a less useful and unwieldy tool due to the limitations in terms of manoeuvrability imposed by the traditional line-of-sight systems that displayed a great difficulty in obtaining sufficient anatomical exposure. Engineering advancements, associated with lower costs, facilitated the reaffirmation of the electrosurgery in laparoscopy and has relegated the laser to limited utilisation in specialised centers (Choussein et al., 2015).

In the recent years, the use of the laser in gynaecological laparoscopy was limited by high cost, low availability and long learning curves (Law et al., 2014).

## Opportunities

In recent years, technological advances and technical improvements are increasing the number of procedures in minimally invasive gynaecology that can exploit laser technology again. Robotic myomectomy, thanks to the advent of a flexible, fully articulated CO_2_ laser delivery system is demonstrated to be feasible and safer than other alternatives such as electrosurgery or ultrasonic scalpel (Sutton et al., 1994).

Lasers delivered by flexible fibres assure both higher incising efficiency and superior wound- healing effects on the uterus than monopolar electrosurgery (Choussein et al., 2015), leading to better obstetrical outcomes in terms of the risk of uterine rupture during pregnancy (Parker et al., 2010).

The higher costs of fully articulated CO_2_ laser delivery systems find their potential justification in the reduction of direct and indirect costs by lowering complication rates and shortening hospital stay.

The CO_2_ laser could also be used to treat women affected by deep infiltrating endometriosis (DIE) in different settings. Meuleman et al. (2011) demonstrated the achievement of low complication rates and a good clinical outcome within 2 years of surgery in a subgroup of women with DIE and colorectal wall invasion treated by a multidisciplinary laparoscopic surgery including CO_2_ laser and consisting of radical endometriosis excision with segmental bowel resection and reanastomosis. Kristensen et al. (2007) confirmed that the laser could be effectively used to treat patients with recto-vaginal pouch and rectovaginal septum endometriosis, with a significant statistical difference between preoperative and postoperative pain scores and quality of life.

Over the years, the laser has also been used in other fields, such as in hysteroscopic surgery. In this field, different kinds of lasers have been used; the Nd-Yag laser, KTP or the Argon laser. The diode laser represents the most significant novelty and is able to produce two wavelengths between 980 and 1470 nm. These wavelengths allow simultaneous cutting and coagulation generating haemostasis significantly higher than the CO_2_ laser. In a pilot study of 18 patients, Nappi et al. (2016) used the diode laser for hysteroscopic metroplasty, demonstrating how the procedure was safe, feasible, potentially preventing the formation of intrauterine adhesions and reducing the risk of recurrences. The same group also demonstrated the safety and effectiveness of the diode-laser hysteroscopic endometrial polypectomy in a prospective study on 300 women (Nappi et al., 2017). A randomised clinical trial has demonstrated how polypectomy with the diode laser resulted in fewer relapses and a higher procedure satisfaction rate compared to the bipolar energy system (Lara- Domínguez et al., 2016). Other recent studies also proposed office hysteroscopic laser enucleation of submucous myomas with good results (Haimovich et al., 2015).

## Threats

The laser showed several technical disadvantages; it can only be used with a rigid lens system, it can be reflected by surgical instruments leading to injury of nontargeted tissues, it also hides the risk of igniting flammable materials or causing eye damage and, particularly with the CO_2_ laser, it is difficult to use in presence of a haemorrhagic field because it is absorbed by water and other fluids (Bailey et al., 2014).

## Concluding Remarks

The laser in gynaecology is indicated for a growing range of minimally invasive surgery procedures. It appears that laparoscopic laser vaporisation is a good option for the treatment of ovarian endometriomas as it reduces the damage to ovarian tissue compared to laparoscopic stripping (Munrós et al., 2019). Moreover, it can be useful in the treatment of DIE in patients with recto-vaginal pouch and rectovaginal septum endometriosis (Kristensen et al., 2007).

The laser has also been proposed for robotic myomectomy, thanks to the advent of a flexible, fully- articulated CO_2_ laser delivery system. In addition, the diode laser has been widely used in hysteroscopic surgery: in case of endometrial polyps it is safe and effective (Nappi et al., 2017); it can be used for metroplasty (Nappi et al., 2016); in recent studies the diode laser has been proposed to treat submucous myomas.

On the other hand, the use of the laser in gynaecological endoscopy has been limited, over the years, by high costs, a low availability and long learning curves (Law et al., 2014).

Technological advancement and cost reduction are necessary to further extend its application in minimally invasive gynaecological surgery.
